# Vitamin C Rescues *in vitro* Embryonic Development by Correcting Impaired Active DNA Demethylation

**DOI:** 10.3389/fcell.2021.784244

**Published:** 2021-11-19

**Authors:** Meiqiang Chu, Fusheng Yao, Guangyin Xi, Jiajun Yang, Zhenni Zhang, Qianying Yang, Jianhui Tian, Lei An

**Affiliations:** Key Laboratory of Animal Genetics, Breeding and Reproduction of the Ministry of Agriculture and Rural Affairs, National Engineering Laboratory for Animal Breeding, College of Animal Science and Technology, China Agricultural University, Beijing, China

**Keywords:** vitamin C, active DNA demethylation, TET dioxygenases, preimplantation embryos, *in vitro* fertilization, lineage differentiation, epigenetic errors

## Abstract

During preimplantation development, a wave of genome-wide DNA demethylation occurs to acquire a hypomethylated genome of the blastocyst. As an essential epigenomic event, postfertilization DNA demethylation is critical to establish full developmental potential. Despite its importance, this process is prone to be disrupted due to environmental perturbations such as manipulation and culture of embryos during *in vitro* fertilization (IVF), and thus leading to epigenetic errors. However, since the first case of aberrant DNA demethylation reported in IVF embryos, its underlying mechanism remains unclear and the strategy for correcting this error remains unavailable in the past decade. Thus, understanding the mechanism responsible for DNA demethylation defects, may provide a potential approach for preventing or correcting IVF-associated complications. Herein, using mouse and bovine IVF embryos as the model, we reported that ten-eleven translocation (TET)-mediated active DNA demethylation, an important contributor to the postfertilization epigenome reprogramming, was impaired throughout preimplantation development. Focusing on modulation of TET dioxygenases, we found vitamin C and α-ketoglutarate, the well-established important co-factors for stimulating TET enzymatic activity, were synthesized in both embryos and the oviduct during preimplantation development. Accordingly, impaired active DNA demethylation can be corrected by incubation of IVF embryos with vitamin C, and thus improving their lineage differentiation and developmental potential. Together, our data not only provides a promising approach for preventing or correcting IVF-associated epigenetic errors, but also highlights the critical role of small molecules or metabolites from maternal paracrine in finetuning embryonic epigenomic reprogramming during early development.

## Introduction

Well-orchestrated epigenomic reprogramming that extensively occurs during the early phases of mammalian development is essential for normal embryogenesis. However, epigenetic events during the critical developmental window, especially by preimplantation stage, are very susceptible to environmental perturbations such as *in vitro* manipulation and culture of embryos, and thus leading to epigenetic errors. Increasing evidence based on epidemiologic analyses and laboratory studies suggested that *in vitro* fertilization (IVF)-induced epigenetic errors were tightly linked to a series of complications, such as embryonic lethality, fetal overgrowth, postnatal disorders, and shortened life span (Halliday et al., 2004; Sutcliffe et al., 2006; Rexhaj et al., 2013; Chen et al., 2015; Tan et al., 2016a; Johnson et al., 2018). Thus, although children born after IVF have exceeded nine millions and contributed to 1–5% of all newborns in developed countries (Manipalviratn et al., 2009), and the great majority of IVF-conceived offspring are in good health, IVF-induced epigenetic risks remain a matter of great concern during the past decades.

Among IVF-associated epigenetic errors, DNA methylation defects are remarkable and extensively studied in humans (Katari et al., 2009; Vermeiden and Bernardus, 2013; Hattori et al., 2019), domestic animals (Deshmukh et al., 2011; Chen Z. et al., 2013; Chen et al., 2015), and mouse models (Li et al., 2005; Rivera et al., 2008; Li et al., 2011). However, the mechanism underlying IVF-associated DNA methylation defects, remains poorly understood. Thus, the effective strategy for preventing or correcting those defects remains lacking. Our recently published study identified IVF embryos undergo impaired *de novo* DNA methylation during implantation and postimplantation stages. We also demonstrated that FGF signaling repression and consistent *Dnmt3b* inhibition could be responsible for this defect, and identified FGF signaling as the main target for correcting IVF-associated DNA methylation defects (Fu et al., 2020). This work, together with our earlier study that identified impaired X chromosome inactivation is responsible for female-biased developmental defects and skewed sex ratio (Tan et al., 2016a), highlight the importance of understanding the mechanism of IVF-associated defects for improving current *in vitro* culture system.

During preimplantation development, one of the most remarkable epigenomic reprogramming events is DNA demethylation that extensively occurs in newly formed embryos following fertilization. DNA demethylation is crucial to establish a hypomethylated genome of the blastocyst, which is essential for regulating pluripotency in the naive epiblast cells (Tan and Shi, 2012; Smith and Meissner, 2013; Messerschmidt et al., 2014). Of note, comprehensive DNA demethylation from the zygote to blastocyst stage largely depends on ten-eleven translocation (TET) proteins TET1, TET2 and TET3 that can oxidize 5mC and generate 5mC derivatives, including 5-hydroxymethylcytosine (5hmC). As the Fe(II) and α-ketoglutarate (α-KG)-dependent dioxygenase, TET proteins require α-KG, oxygen and Fe(II) for their enzymatic activity. Thus, small molecules that regulates TET enzymatic activity, such as α-KG and vitamin C that can maintain reduced Fe(II) (Kohli and Zhang, 2013), are critical for fine-tuning the prosses of active DNA demethylation (Minor et al., 2013; Yin et al., 2013).

Evidences from mice embryos of genetic depletion of individual *Tet* or in combination give rise to increased embryonic lethality throughout pregnancy, as well as developmental defects that can be observed as early as the 2-cell stage (Ito et al., 2010; Gu et al., 2011; Kang et al., 2015) Despite the important role of TET-mediated DNA demethylation by preimplantation stage in determining embryonic survival and growth, previous results observed in rat and porcine IVF embryos (Yoshizawa et al., 2010; Deshmukh et al., 2011), as well as our analyses from mice and bovine, suggested impaired DNA demethylation in IVF preimplantation embryos. However, the underlying regulatory mechanism have yet to be functionally elucidated, and the strategy for correcting this error remains unavailable until now.

In the present study, focusing DNA hypermethylation in IVF blastocysts, we used mouse and bovine IVF embryos as the model and reported that TET-mediated active DNA demethylation is impaired throughout preimplantation development. Detections of *in vivo* conceived preimplantation embryos and their maternal oviductal environment indicated that the requirement for vitamin C (also known as l-ascorbic acid or l-acerbate), the important co-factors for stimulating TET enzymatic activity, during preimplantation development would be satisfied by both oviductal paracrine and embryonic autocrine. Accordingly, we identify the impaired active DNA demethylation can be corrected by incubation of IVF embryos with vitamin C, and thus improving their lineage differentiation and developmental potential. Thus, our data not only provides a promising approach for preventing or correcting IVF-associated epigenetic errors, but also highlights the critical role of maternal paracrine in finetuning embryonic epigenomic reprogramming during early development.

## Materials and Methods

### Animals

ICR female mice aged 8 weeks, male mice aged 10 weeks were kept in controlled conditions of temperature (24°C) and light (12 h light:12 h dark) and had free access to food and water. All mice were approved by the Institutional Animal Care and Use Committee of China Agricultural University.

### *In vivo* (IVO) Embryo Collection

The female mice were superovulated by intraperitoneal injection of 5 IU of pregnant mare serum gonadotropin (PMSG, Ningbo, China) and a further intraperitoneal injection 48 h of 5 IU human chorionic gonadotrophin (HCG, Ningbo, China). The female mice were cocaged individually with male mice after the hCG injection. On the next morning, the females with vaginal plug were selected as mating successfully. Zygotes, the 2-cell, 4-cell, 8-cell embryos, morulae and blastocysts (16–20 h, 44–46 h, 54–56 h, 66–68 h, 74–76 h and 94–96 h post HCG, respectively) were recovered from donors by flushing the oviduct and uterus with M2 medium.

### *In vitro* Fertilization, Embryo Culture and Embryo Collection

The IVF procedure were performed as previously described (Ren et al., 2015; Tan et al., 2016a; Tan et al., 2016b; Ren et al., 2017). In brief, sperm were released from the cauda epididymis and capacitated for 1 h in modified Krebs-Ringer bicarbonate medium (TYH), and the cumulus-oocyte complexes were transferred into modified human tubal fluid (mHTF) for 30 min, then inseminated for 4 h. After the insemination, zygotes were washed and cultured in potassium simplex optimized medium containing amino acids (KSOM + AA; Millipore, Darmstadt, Germany) at 37°C in 5% CO_2_. IVF embryos at different preimplantation stages were collected based on their developmental progress and morphology.

### Preparation of Bovine Blastocysts

Bovine oocytes were collected from ovaries obtained from a slaughterhouse, and matured in Tissue Culture Medium-199 (TCM-199, Thermo Fisher Scientific, Rockford, IL, United States) plus 10% (vol/vol) FBS (HyClone, Marlborough, United States), 1% antibiotic-antimycotic (Gibco BRL, Thermo Fisher Scientific), and 10 ng/ml epidermal growth factor (22–24 h). *In vitro* fertilization was conducted in Bracket and Oliphant’s (BO) medium. Briefly, matured oocytes with multiple layers of expanded cumulus cells were washed and then in BO fertilization medium supplemented with 6 mg/ml essential fatty acid-free (FAF)-BSA (Millipore, Billerica, MA, United States) and 10 mg/ml heparin. Fifteen to 20 cumulus-oocyte complexes (COCs) were placed in 50 μL BO medium, under mineral oil, containing frozen-thawed sperm (1–2 × 10^6^ sperm/ml) for 24 h in 5% (vol/vol) CO_2_ in air at 38.5°C. Cumulus cells were removed by pipetting, and presumptive zygotes were cultured in 20-μl drops of Bovine VitroCleave (IVF Vet Solutions, North Adelaide, Australia) under mineral oil for 5 days. On day 5, embryos were transferred in groups of 5–10–20-μl drops of Bovine VitroBlast (IVF Vet Solutions) under mineral oil.

### Extraction of Oviductal/Uterine Fluids and Preparetion of Oviductal/Uterine Tissue

Oviduct and uterine fluids were collected from female mouse according to the protocol of a previous study (Harris et al., 2005). Briefly, once oviduct excised, the tissue was dried and placed under mineral oil, stabbed by needle. Then we collected the fluids with mouse pipette into 1.5 ml tube. Uterine was ligatured with nylon thread, gentle pressured from the thread end to another one, the fluids were flowed into 1.5 ml tube. All fluids were centrifuged for 5 min at 12,000 revolutions per minute (rpm) to obtain the supernatants, stored into -80°C. Remained tissue was washed twice with PBS solution, then 0.1 g tissue was put into 1.5 ml tube and immediately throwed in liquid nitrogen until assay.

### RNA Extraction and Quantitative Real-Time PCR

Total RNA was extracted from embryos, the oviduct and uterus with TRIzol (Thermo Fisher Scientific) following the manufacturer’s instructions. Then the reverse transcription was performed by the Hiscript R Ⅱ Q RT Supermix (Vazyme, Nanjing, China) according to the manufacturer’s instructions. Quantitative real-time PCR (qRT-PCR) was performed with SoFast EvaGreen Supermix (BioRad, Hercules, California, United States) using a CFX96 real-time PCR machine (BioRad). All primers were listed in Supplementary Table S1.

### RNA–Seq of Embryos and Tissue

Total RNAs were extracted from embryos, oviducts and uterine at different stages with TRIzol Reagent (Invitrogen, Carlsbad, CA, United States). Then the RNA was delivered to BGI (BGI, Shenzhen, China) for sequencing. Gene expression levels were measured in reads per kilobase of exon model per million mapped reads (RPKM). The RPKM was listed in Supplementary Table S2–4.

The database for Annotation, Visualization and Integrated Discovery (DAVID v6.7; http://david.abcc.ncifcrf.gov) was used to annotate biological themes (gene ontology, GO). The Kyoto Encyclopedia of Genes and Genomes (KEGG; http://www.genome.jp/kegg/) was used to determine the associated pathways. Phenotype annotations were analyzed based on the Mouse Genome Informatics (MGI; http://www.informatics.jax.org/phenotypes.shtml) database.

### Immunofluorescence Analysis

Collected embryos were washed three times with 0.1%PVA-PBS and then washed with acidic Tyrode’s solution to eliminate the zona pellucida. Then embryos were fixed in 4% paraformaldehyde in PBS overnight at 4°C. After permeabilized with 0.5% Triton X-100 in 0.1% PVA-PBS, embryos were blocked in 1% BSA (Millipore) in 0.1% PVA-PBS for 1 h, then sequentially incubated with primary antibody overnight at 4°C. Next, the embryos were washed three times with 0.1%PVA-PBS for 5 min and incubated with secondary antibodies for 1 h at room temperature. Finally, the samples were treated with DAPI for 5 min, mounted with coverslips. Images were recorded using fluorescence microscope (BX51TRF; Olympus, Tokyo, Japan) and processed using ImageJ software (Rawak Software Inc., Stuttgart, Germany).

For 5mC and 5hmC staining, nuclear DNA was denatured with 4M HCl for 10 min, neutralized with Ph8.0 Tris-HCl for 15 min, then embryos were blocked overnight, incubated with primary antibody for 2 h. Other steps were same as described above. The following antibodies used in this research were listed as follows: anti-5mC (1:200, Active motif 39,649), anti-5hmC (1:500 dilution, 39,791, Active motif, California, United States), anti-TET1 (1:200 dilution, GTX124207, GeneTex, Irvine, CA, United States), anti-TET2 (1:100 dilution, ab94580, Abcam, Cambridge, UK), anti-NANOG (1:500 dilution, ab80892), anti-CDX2 (1:500 dilution, BioGenex-MU392A-UC, BioGenex Laboratories, Fremont, CA 94538, United States).

### Vitamin C Content Assay

Preimplantation embryos, the oviduct and uterus, as well as the oviductal or uterine fluid were prepared as described above. Each 50 oocytes, 50 embryos or total cumulus cells surrounding 150 oocytes as a biological replicate, CC indicates the total cumulus cells surrounding one oocyte. The ascorbic acid assay kit (ab65346, Abcam) was used for detecting the vitamin C content according to the manufacturer’s instructions.

### Blastocyst Transfer and E7.5 Embryos Photographic

Pseudo-pregnant female mice (recipients) were co-caged individually with vasectomized males 3.5 days before embryo transfer. The morning after mating, the recipients were checked for the presence of a vaginal plug. The day of plugging was considered as day 0.5 of the pseudo-pregnancy. 12 well-developed blastocysts were transferred into each recipient. At embryonic day (E)7.5 (4 days of embryo transfer), the conceptuses covered with decidual mass were gently teased away from the uterus, E7.5 embryos were E7.5 embryos were separated and washed in PBS, then imaged using a stereomicroscope (SZX16; Olympus, Tokyo, Japan) equipped with a digital camera.

### Statistical Analysis

Student’s t-test or one-way ANOVA were used to analyze the difference among groups by using SPSS 23.0 software (Statistical Package for the Social Sciences). Statistically significant differences were defined as *p* < 0.05.

## Results

### IVF Embryos Undergo Impaired Active DNA Demethylation and Exhibit Global Hypermethylation by Blastocyst Stage

**FIGURE 1:**
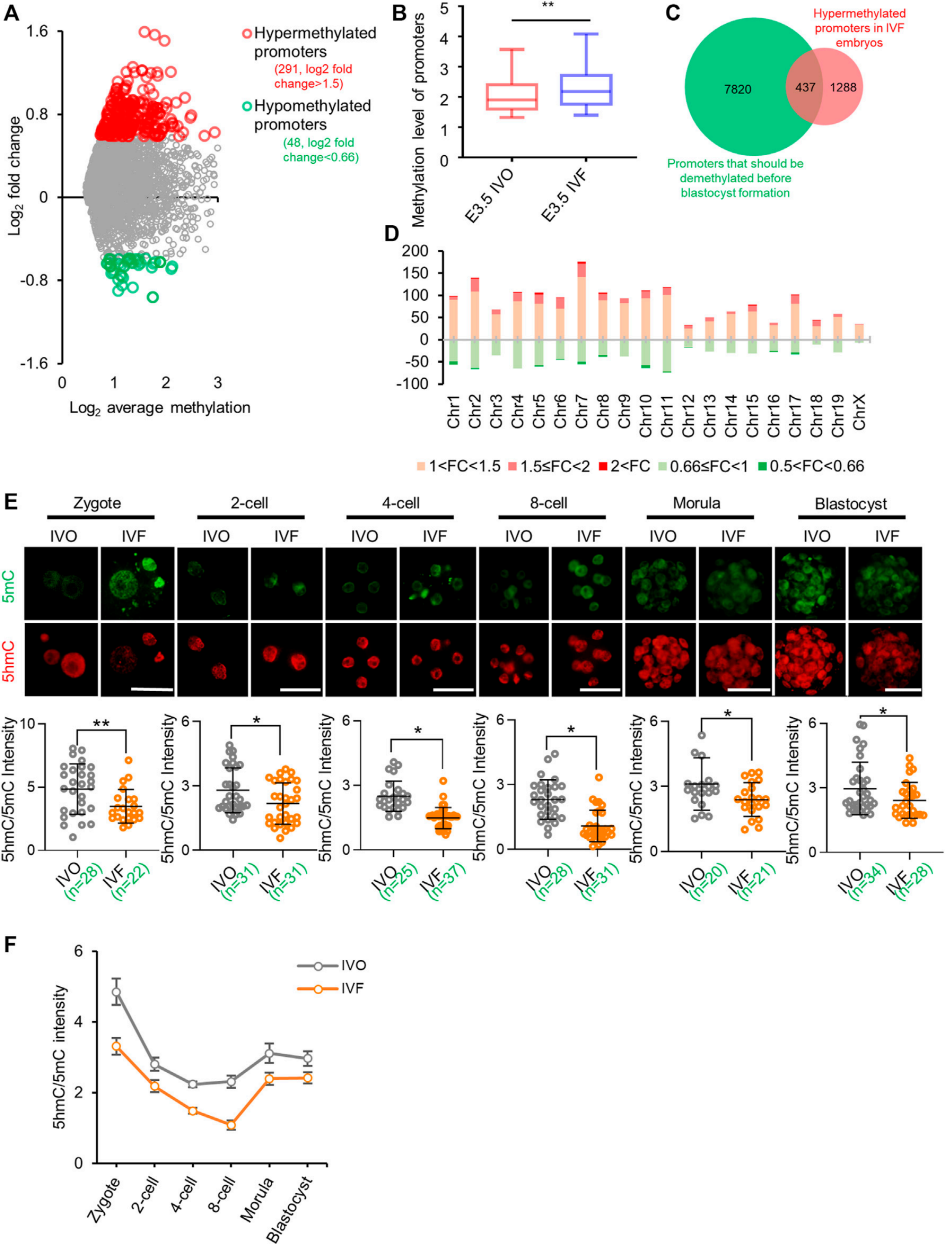
IVF embryos undergo impaired active DNA demethylation and exhibit global hypermethylation by blastocyst stage **(A)** Distribution of differentially methylated promoters in IVF blastocysts compared with their IVO counterparts **(B)** Box plot of promoter methylation levels of IVO and IVF blastocysts **(C)** The Venn diagram of hypermethylated promoters in IVF blastocysts (Ren et al., 2015) and promoters that should be demethylated before blastocyst formation (Smith et al., 2012) **(D)** The chromosome-wide distribution of hypermethylated and hypomethylated promoters with different fold changes in IVF blastocysts **(E)** Immunofluorescent images of 5mC (green) and 5hmC (red) staining in IVO and IVF embryos from zygote to blastocyst stages. Lower panels: quantification of 5hmC/5mC ratio in IVO and IVF embryos. Circles represent the relative ratio in each embryo. The number of embryos in each group is indicated **(F)** Dynamics of TET-mediated active demethylation in IVO and IVF preimplantation embryos, revealed by 5hmC/5mC ratio at each stage. Data show the means ± SD of three independent experiments. *p < 0.05, **p < 0.01. Scale bar, 50 μm.

To test the impact of IVF processes on DNA demethylation during preimplantation development, we compared our previously published global MeDIP-seq data of IVO and IVF blastocysts (Ren et al., 2015; Ren et al., 2017). Focusing on promoter DNA methylation, which undergoes postfertilization demethylation (Smith et al., 2012) and participates in TET-induced transcriptional regulation (Ito et al., 2010; Gu et al., 2011; Blaschke et al., 2013), we found IVF blastocysts showed higher DNA methylation levels **(** Figure 1A **)** and a greater proportion of promoters were relatively hypermethylated in IVF blastocysts compared with their IVO counterparts **(** Figure 1B **)** Similarly, reanalysis of previously published DNA methylation array data also showed more hypermethylated regions in IVF bovine blastocysts (Supplementary Figure S1A). In addition, Gene ontology (GO) analysis and Mouse Genome Informatics (MGI)-based phenotype annotations suggested hypermethylated genes can participate in many basic molecular functions and cellular processes, and are essential for normal embryonic development and survival throughout the pregnancy (Supplementary Figure S1B, C). Venn diagram based on previously published methylome (Smith et al., 2012) showed that a substantial proportion of promoters that should be demethylated before blastocyst formation, were hypermethylated promoters in IVF blastocysts (Figure 1C). Next, we detected the chromosome-wide distribution of hypermethylated promoter in IVF blastocysts, and found hypermethylated promoters were globally distributed across all autosomes and sex chromosomes (Figure 1D). These results suggest that IVF preimplantation embryos may undergo extensive impairment in DNA demethylation.

Given results of an early study suggested that impaired DNA demethylation can be initially observed in paternal genome of IVF zygotes (Yoshizawa et al., 2010), we speculated that TET-mediated active demethylation may be impaired. To confirm this hypothesis, we tested 5mC and 5hmC levels at each stage of preimplantation development. Quantitation of 5hmC/5mC ratio indicated that TET-mediated active DNA demethylation was consistently impaired in IVF preimplantation embryos (Figures 1E,F).

### Expression of *Tet* Family Members is Inhibited in IVF Preimplantation Embryos

**FIGURE 2:**
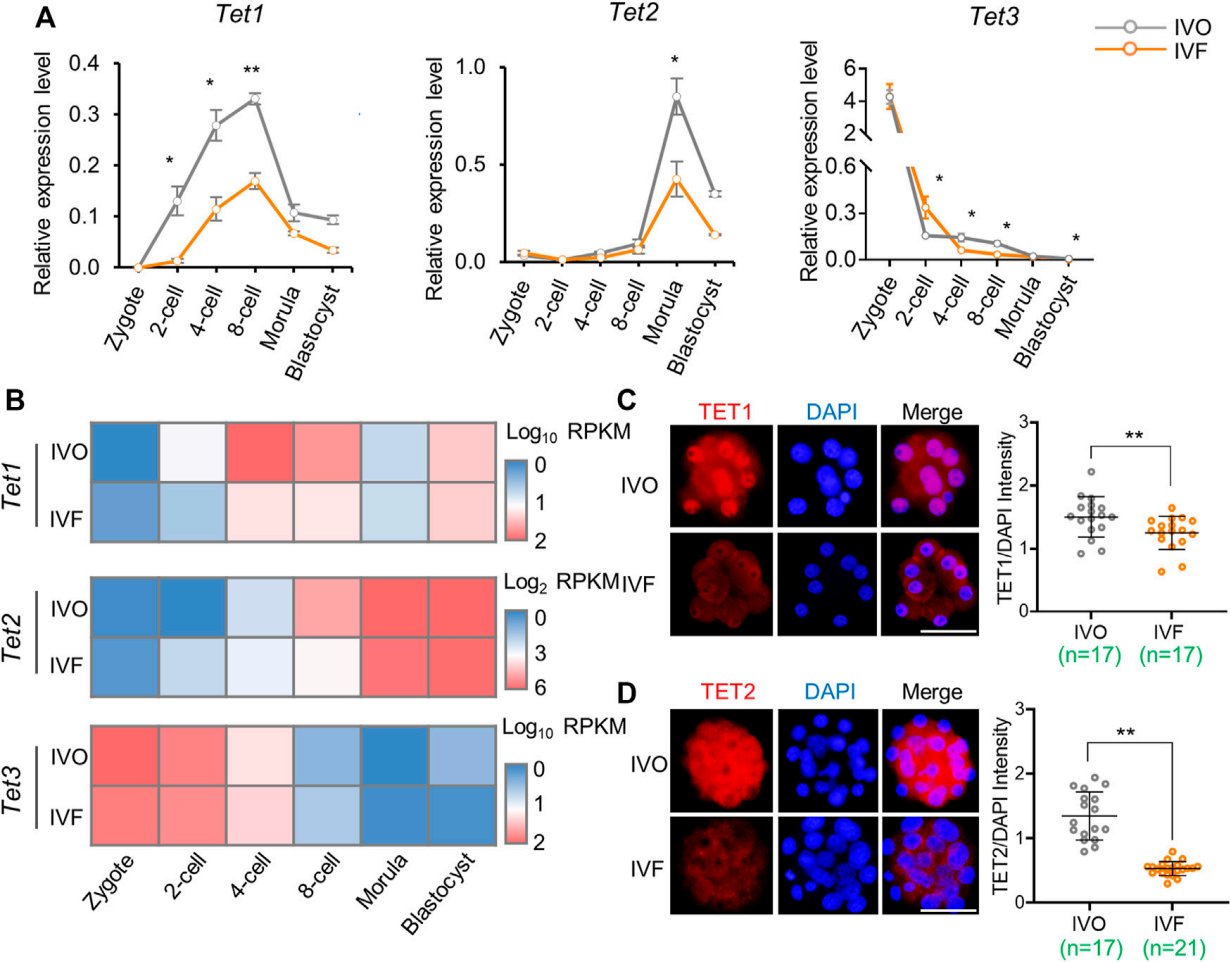
Expression levels of *Tet* family members is inhibited in IVF preimplantation embryos **(A)** Relative expression levels of *Tet1*, *Tet2,* and *Tet3* in IVO and IVF embryos detected by qRT-PCR during preimplantation development **(B)** Heat map illustrating expression patterns of *Tet1*, *Tet2,* and *Tet3* in IVO and IVF embryos detected by RNA-seq during preimplantation development **(C–D)** Immunofluorescent images of TET1 **(C)** and TET2 **(D)** in IVO and IVF 8-cell embryos and morulae, respectively. Right panels in C and D: quantification of the relative protein levels of TET1 and TET2. Each circle represents the level of immunofluorescent signal of TET1 or TET2 relative to the DAPI. The number of embryos in each group is indicated. Data show the means ± SEM **(A)** and means ± SD **(C–D)** of three independent experiments. At least 50 embryos were pooled at each time point for each replicate of qRT-PCR detection. *p < 0.05, **p < 0.01. Scale bar, 50 μm.

Having confirmed the defects of TET-mediated active DNA demethylation in IVF embryos, we next asked if the gene expression level of *Tet* family members was inhibited in IVF preimplantation embryos. We found *Tet1* and *Tet2* expression, which should be increased during cleavage stages, were significantly inhibited in IVF embryos at the two- to 8-cell stage and the morula stage respectively (Figure 2A). In addition, *Tet3* expression, although showed maternal deposition, was consistently inhibited in IVF embryos from the 4-cell stage onwards (Figure 2A). The IVF-induced expression inhibition of *Tet* family members was further confirmed using our RNA-seq data (Figure 2B). Moreover, the inhibition of TET1 and TET2 were also validated on the protein level. In line with the result of mRNA detection, quantitation of immunofluorescence signal indicated that TET1 and TET2 proteins were significantly deficient in IVF 8-cell embryos and morulae, respectively (Figures 2C,D). Of note, we also noticed that a proportion of blastomeres exhibit cytoplasmic localization of TET2, and the mislocation in IVF embryos were more evident (Figure 2D).

Next, we attempted to test if the inhibited expression of *Tet* family members in IVF embryos could be rescued by supplementing cytokines or small molecules that have been reported to upregulate *Tet* expression: retinoic acid (Hore et al., 2016), FGF2 (Choi et al., 2018), LIF (Tahiliani et al., 2009; Koh et al., 2011) and insulin (Lv et al., 2017). Given *Tet3* is a well-known maternally deposited transcripts, we next focused on the embryo-expressed *Tet1* and *Tet2*. However, neither these factors alone (Supplementary Figure S2A–D) nor combinations (Supplementary Figure S2E, F **)** could upregulate expression levels of *Tet1* and *Tet2* in IVF embryos.

### TET Cofactors Vitamin C and α-ketoglutarate Are Enriched in Oviductal Environment

**FIGURE 3:**
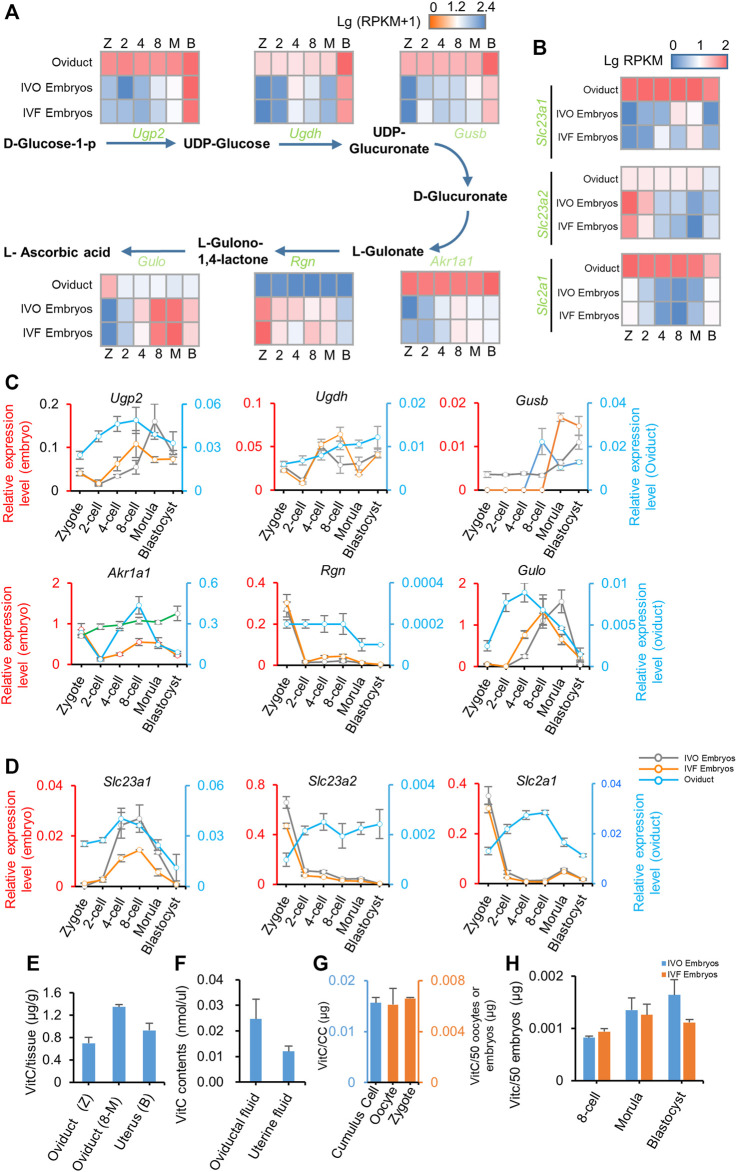
Vitamin C is enriched in oviductal environment **(A, B)** Heat map illustrating the expression patterns of genes underlying vitamin C biosynthesis **(A)** and transfer **(B)** pathway in IVO and IVF embryos, as well as in temporally corresponding oviduct detected by RNA-seq during preimplantation development **(C, D)** Relative expression levels of genes underlying vitamin C biosynthesis **(C)** and transfer **(D)** pathway in IVO and IVF embryos (left axis), as well as in temporally corresponding oviduct (right axis) detected by qRT-PCR during preimplantation development **(E-H)** Detection of vitamin C contents in oviduct/uterus **(E)** and oviductal/uterine fluid **(F)**, oocytes, zygotes and surrounding cumulus cells **(G)**, as well as IVO and IVF preimplantation embryos **(H)** at different stages. CC indicates the total cumulus cells surrounding 50 oocytes. Data shows the means ± SEMs of three independent experiments. At least 50 embryos were pooled in each group for each replicate of qRT-PCR detection.

Having failed to rescue IVF-induced *Tet* inhibition, we next asked if impaired active DNA demethylation can be rescued by enhancing TET activity. To this end, we focused on vitamin C and α-ketoglutarate (α-KG) because TET enzymes are Fe(II)- and α-KG-dependent dioxygenases, and vitamin C can stimulate TET activity by maintaining reduced Fe(II) (Kohli and Zhang, 2013). Time-course expression profiling of genes related to vitamin C synthesis and transport in preimplantation embryos, as well as in temporally corresponding oviduct based on our RNA-seq data (Figures 3A,B), indicated that vitamin C synthesis and transport occurred in both preimplantation embryos and the oviduct, especially in the oviduct. These findings were further supported by results of qRT-PCR (Figures 3C,D). More importantly, we detected that vitamin C was highly enriched in the oviduct and uterus throughout the preimplantation stage (Figure 3E), and thus being detectable in the oviductal and uterine fluid (Figure 3F). In addition, we also detected low-level vitamin C in preimplantation embryos and found vitamin C might be pre-deposited in oocytes, and no significant difference can be detected between IVO and IVF embryos (Figures 3G,H). These results suggest that the requirement for vitamin C during preimplantation development may be satisfied by both oviductal paracrine and embryonic autocrine.

Similarly, our results also suggest that the requirement for α-KG, another cofactor for TET dioxygenases, may be also satisfied via synergistic effect of oviductal paracrine and embryonic autocrine, because α-KG synthetic and transporter genes were detectable in both embryos and the oviduct by preimplantation stage (Supplementary Figure S3A–C). Collectively, these results led us to test if supplementation of embryo culture medium with vitamin C and/or α-KG could rescue impaired active DNA demethylation in IVF embryos.

### Vitamin C, but Not α-KG, Enhances TET Enzymatic Activity in IVF Preimplantation Embryos

**FIGURE 4:**
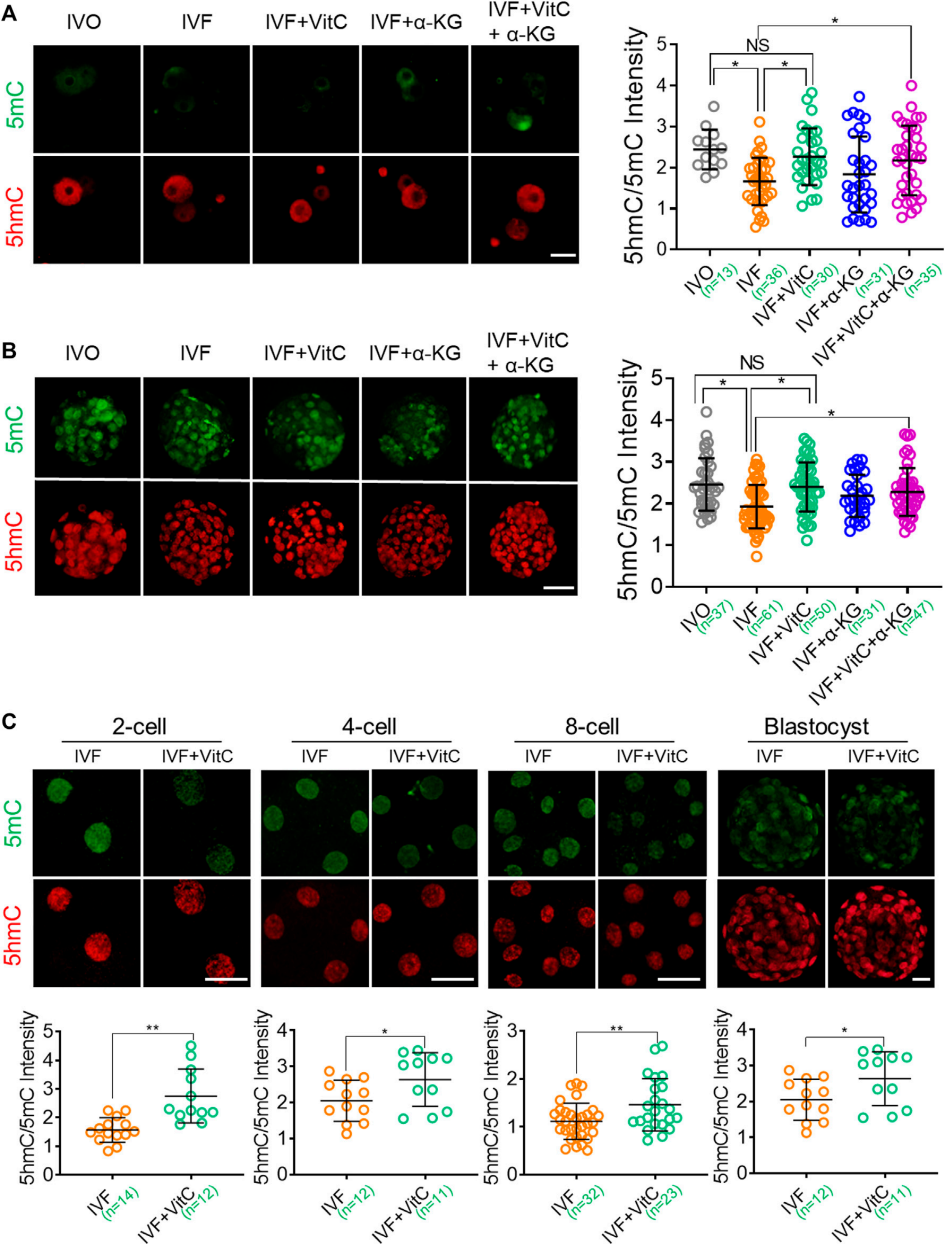
Vitamin C enhances TET enzymatic activity in IVF preimplantation embryos **(A, B)** Effects of supplementation with vitamin C or α-KG alone, or in combination on active DNA demethylation in IVF zygotes **(A)** and blastocysts **(B)**. Left panels in A and B: Immunofluorescent images of 5mC (green) and 5hmC (red) staining in embryos in each group. Right panels in A and B: quantification of 5hmC/5mC ratio in IVF embryos in the control group or treatment groups. Circles represent the relative ratio in each embryo. The number of embryos in each group is indicated **(C)** Effects of vitamin C supplementation on active DNA demethylation in bovine IVF preimplantation embryos. Lower panels in C: quantification of 5hmC/5mC ratio in bovine IVF embryos in the control group or treatment group. Circles represent the relative ratio in each embryo. The number of embryos in each group is indicated. Data shows the means ± SD of three independent experiments. *p < 0.05, **p < 0.01. Scale bar, 50 μm.

Next, we screened effective concentration of vitamin C supplementation by evaluating its efficacy in prompting preimplantation development, because TET deficiency would impair survival and growth of preimplantation embryos (Kang et al., 2015). We found 100 μg/ml vitamin C supplementation to culture medium significantly increased cleavage rate and blastocyst rate (Supplementary Figure S4A, B). Using this concentration, we found vitamin C supplementation significantly enhanced TET enzymatic activity in IVF embryos throughout the preimplantation development to the levels comparable to those of IVO embryos, as revealed by increased 5hmC/5mC ratio in both zygotes (Figure 4A and blastocysts (Figure 4B). Moreover, the beneficial effect of vitamin C supplementation on enhancing TET enzymatic activity was also confirmed using bovine IVF embryos as the model (Figure 4C), although the expression patterns of bovine *TET* enzymes were largely distinct from those in mouse embryos (Jiang et al., 2014; Salilew-Wondim et al., 2015) (Supplementary Figure S4F, G).

In contrast, however, α-KG supplementation using previously published effective concentration (Zhang et al., 2019) just tended to, but not significantly, enhance TET enzymatic activity in both IVF zygotes and blastocysts. In addition, combined supplementation of vitamin C and α-KG didn’t display synergistic effect on prompting TET enzymatic activity (Figure 4A, B), in line with their effect on preimplantation development (Supplementary Figure S4C–E).

### Vitamin C Regulates Preimplantation Lineage Differentiation and Promotes Developmental Potential of IVF Blastocysts

**FIGURE 5:**
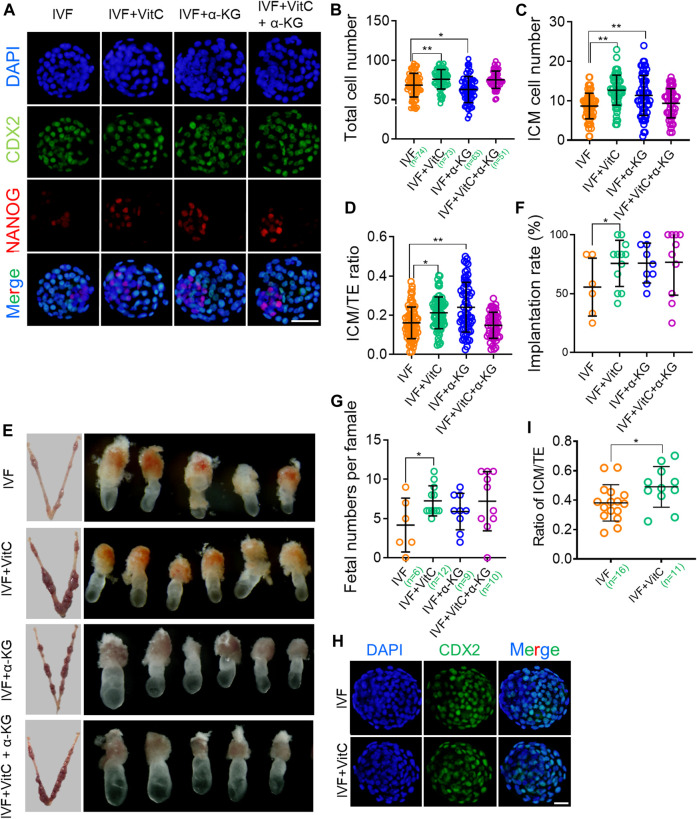
Vitamin C regulates preimplantation lineage differentiation and promotes developmental potential of IVF blastocysts **(A)** Immunofluorescent images of NANOG and CDX2 in IVF blastocysts incubated with vitamin C or α-KG alone, or in combination. The embryos were counterstained with DAPI **(B-D)** Effects of supplementation with vitamin C or α-KG alone, or in combination on total cell numbers **(B)**, ICM cell numbers **(C)**, and ratios of ICM/TE **(D)** in IVF blastocytes. ICM and TE cell numbers were calculated by counting NANOG-positive and CDX2-positive cells. Circles represent cell numbers or ratios in each embryo. The number of embryos in each group is indicated **(E)** Representative images of the implantation sites (left column) at E7.5 in each group following embryo transfer, and morphological comparison of recovered E7.5 embryos among groups (right column) **(F)** Quantifications of implantation rates of transferred embryos in each group. Circles represent implantation rate of each female recipient **(G)** Number of survived embryos with normal morphologies at E7.5 in each group following embryo transfer. Circles represent survived embryo in each female recipient. The number of recipients in each group is indicated **(H)** Immunofluorescent images of CDX2 in IVF bovine blastocysts incubated with vitamin C **(I)** Effects of supplementation with vitamin C on ratios of ICM/TE in IVF bovine blastocysts. ICM and TE cell numbers was calculated by counting CDX2-negative and CDX2-positive cells. The number of embryos in each group is indicated. Data show the means ± SD of three independent experiments. *p < 0.05, **p < 0.01. Scale bar, 50 μm.

Given previous studies have demonstrated that TET-mediated active DNA demethylation participate in inner cell mass (ICM) specification, and regulate embryonic growth and developmental potential (Ito et al., 2010; Kang et al., 2015), we next tested whether vitamin C-prompted TET enzymatic activity would affect total cell number and lineage differentiation of IVF blastocysts. In addition, we also evaluated the effect of vitamin C supplementation on embryonic developmental potential by detecting implantation rate and postimplantation survival rate following embryo transfer. Our results showed IVF blastocysts exposed to vitamin C displayed a significant increase in total cell number (Figures 5A,B), and ICM cell number (Figures 5A,C). Of note, vitamin C supplementation resulted in a changed lineage differentiation towards the ICM fate in IVF blastocysts (Figures 5A,D). Correspondingly, in comparison to their control counterparts, IVF embryos exposed to vitamin C had significantly higher implantation rate and survival rate shortly after implantation (Figures 5E,F). Similarly, the beneficial effects of vitamin C supplementation on preimplantation lineage differentiation and developmental potential, were also confirmed in bovine IVF embryos (Figures 5H,I).

Moreover, we found α-KG alone, but not in combination with vitamin C, enhanced total cell number and ICM specification of IVF blastocysts, as well as subsequent implantation success (Figures 5A–F). These beneficial effects, were not completely in line with quantification results of 5hmc/5mC ratio (Figures 4A–D), implying that functions of α-KG in improving IVF embryo development are complicated and may be partially independent of TET enzymatic activity.

### The Beneficial Effects of Vitamin C on IVF Embryos are Mediated by TET Enzymatic Activity

**FIGURE 6:**
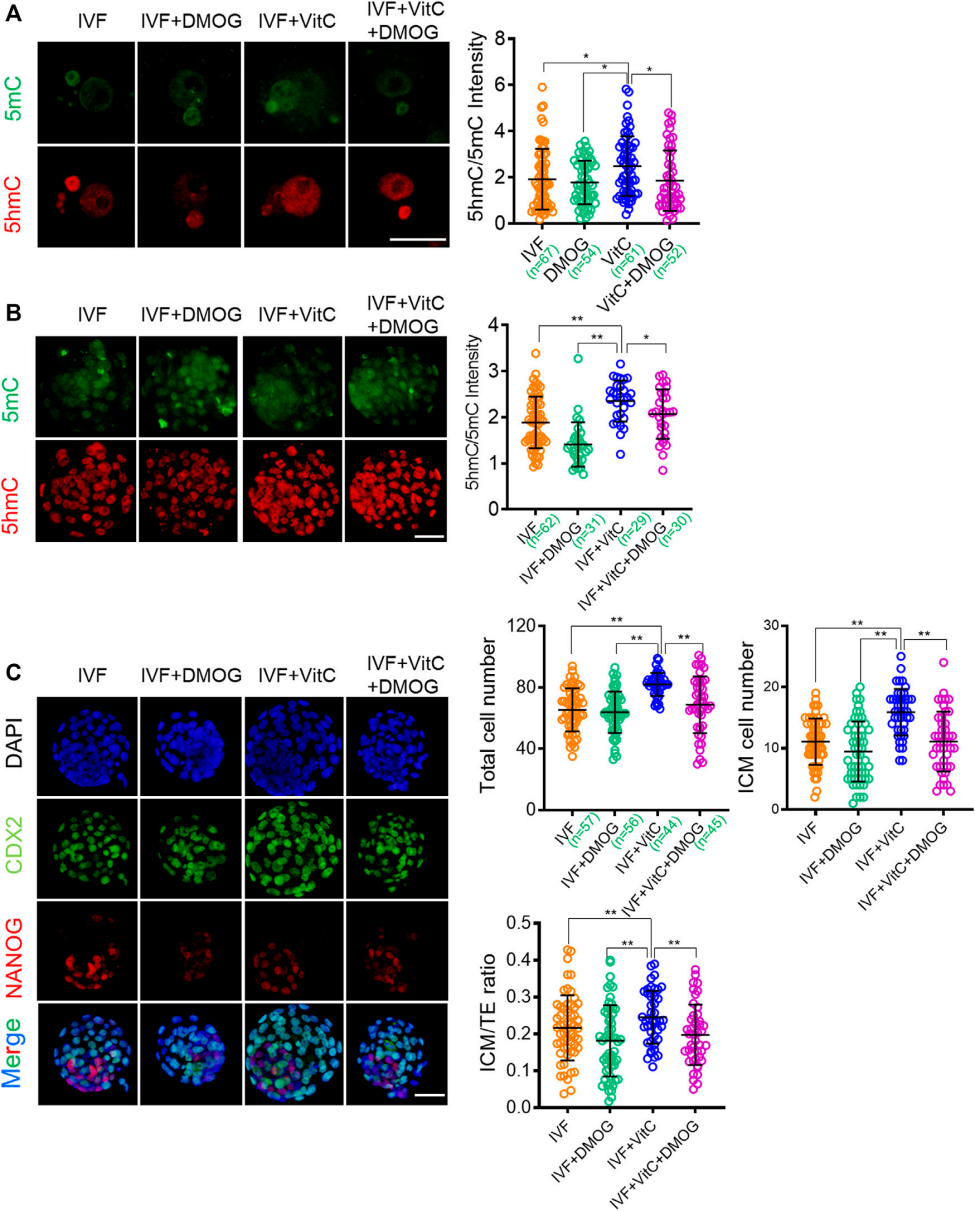
The beneficial effects of vitamin C on IVF embryos are mediated by TET enzymatic activity **(A, B)** Effect of supplementation with vitamin C or TET inhibitor DMOG alone, or in combination on active DNA demethylation in IVF zygotes **(A)** and blastocysts **(B)**. Left panels in A and B: Immunofluorescent images of 5mC (green) and 5hmC (red) staining in embryos in each group. Right panels in A and B: quantification of 5hmC/5mC ratio in IVF embryos in the control group or treatment groups. Circles represent the relative ratio in each embryo. The number of embryos in each group is indicated **(C)** Immunofluorescent images of NANOG and CDX2 in IVF blastocysts incubated with vitamin C or DMOG alone, or in combination. The embryos were counterstained with DAPI **(D-F)** Effect of supplementation with vitamin C or DMOG alone, or in combination on total cell numbers **(D)**, ICM cell numbers **(E)**, and ratios of ICM/TE **(F)** in IVF blastocytes. Data show the means ± SD of three independent experiments. *p < 0.05, **p < 0.01. Scale bar, 50 μm.

Having confirmed the function of vitamin C on rescuing impaired active DNA demethylation in IVF embryos, we next attempted to determine if this beneficial effect was mediated by TET proteins. To this end, we used dimethyloxallyl glycine (DMOG), an inhibitor that blocks TET enzymatic activity (Zhang et al., 2017; Duforestel et al., 2019), to assess the role of TET enzymes in mediating vitamin C-induced active DNA demethylation and developmental advantages. We found DMOG significantly attenuated the active DNA demethylation-prompting effect of vitamin C in both IVF zygotes (Figure 6A) and blastocysts (Figure 6B). In addition, the beneficial effects of vitamin C on total cell number and lineage differentiation of IVF blastocysts were also attenuated by DMOG supplementation (Figure 6C). These results suggest that the beneficial effects of vitamin C are largely mediated by TET enzymatic activity.

## Discussion

DNA methylation in mammalians is an essential epigenetic mark to diverse processes, including transcriptional regulation, protection of genomic integrity, maintenance of gene imprinting and X-chromosome inactivation, as well as repression of transposable elements (Maher, 2005; Manipalviratn et al., 2009; Vermeiden and Bernardus, 2013). During early development, DNA methylation is highly dynamic and susceptible to environmental factors. Fine-tuned DNA methylation dynamics is critical for normal development, whereas any disruption in the dynamics may compromise embryogenesis or lead to long-term complications. Therefore, IVF-associated DNA methylation defects were thought to be linked to development disorders and postanal defects observed in both humans and animals (Manipalviratn et al., 2009; Chen Z. et al., 2013; Shechter-Maor et al., 2018).

To the best of our knowledge, the first case of IVF-associated hypermethylation in preimplantation embryos was reported in rats as early as 2010 (Yoshizawa et al., 2010). This phenomenon has been confirmed repeatedly in porcine IVF blastocysts (Deshmukh et al., 2011). Together with these findings, our results observed form mouse IVF blastocytes, as well as reanalysis of published DNA methylation array data of IVF bovine blastocysts, suggested that IVF-induced hypermethylation in IVF preimplantation embryos might be common among various species, although human embryos cannot be measured *in vivo* until now.

DNA demethylation is a hallmark epigenomic event during preimplantation development, and is essential for normal embryogenesis. It is generally believed that DNA demethylation contributes zygotic gene activation and is dispensable for maintaining the consistency of gene transcription during preimplantation development, which is critical for initiation of nuclear reprogramming towards pluripotency (Bhutani et al., 2010; Shen et al., 2014; Kang et al., 2015). Although it has been reported that replication-dependent DNA dilution, also known as passive demethylation, is the major contributor to DNA demethylation after fertilization (Guo et al., 2014; Shen et al., 2014), TET-mediated active DNA demethylation also plays an important role in epigenetic reprogramming. Knockout of *Tet1* or *Tet3* alone, or in combination, led to attenuated zygotic gene activation, increased cleavage arrest, blastomeres apoptosis or fragments, skewed lineage differentiation in preimplantation embryos, as well as smaller embryonic size and morphological abnormalities during postimplantation development (Ito et al., 2010; Gu et al., 2011; Peat et al., 2014; Kang et al., 2015). These phenotypes, are similar with those observed in IVF embryos (Nie et al., 2013; Ren et al., 2015; Tan et al., 2016a; Tan et al., 2016b), implying the possible involvement of impaired DNA demethylation in IVF-induced developmental defects. This notion is also supported by the development-prompting effect of vitamin C that depends on TET enzymatic activity.

Our results of 5mC and 5hmC staining indicated that TET-mediated active DNA demethylation was impaired in both mouse and bovine IVF embryos throughout preimplantation development. Although TET enzymes displayed evident transcriptional inhibition, our efforts to sitmulate *Tet* expression using cytokines or small molecules that have been reported to upregulate *Tet* expression in other cell types (Tahiliani et al., 2009; Koh et al., 2011; Hore et al., 2016; Lv et al., 2017; Choi et al., 2018), has failed. These results suggest that transcriptional regulation of *Tet* family members in preimplantation embryos, may be partially distinct form that in somatic cells, and need to be further explored in future studies.

Despite this, our study provides an alternative and efficient strategy for rescuing TET-mediated active DNA demethylation in IVF embryos. By supplementing vitamin C to culture medium, we successfully rescued 5mC to 5hmC conversion to levels comparable to those in naturally conceived embryos. In addition to its well-known function as an antioxidant, vitamin C is a well-established cofactor for many Fe(II) and a-KG-dependent dioxygenases, which include collagen prolyl hydroxylases and epigenetic enzymes of histone and DNA methylation (Lu et al., 2015). Among these, TET proteins are key epigenetic enzymes that play pivotal roles in epigenetic remodeling of stem cells and preimplantation embryos (Ito et al., 2010; Gu et al., 2011; Wu et al., 2011; Kang et al., 2015) Vitamin C, as the cofactor of dioxygenases enzymes, has been reported to directly enhance TET enzymatic activity by maintaining reduced Fe(II), and thus simulating TET-mediated active DNA demethylation (Minor et al., 2013; Yin et al., 2013). Based on this mechanism, vitamin C showed ability to induce a blastocyst-like pluripotency in ES cells (Blaschke et al., 2013) and facilitate somatic cell reprogramming (Esteban et al., 2010; Chen J. et al., 2013) via a TET-dependent mechanism.

Given TET-mediated active DNA demethylation plays critical role in regulating preimplantation lineage differentiation and acquiring developmental potential (Ito et al., 2010; Gu et al., 2011; Kang et al., 2015), we also focused developmental phenotypes of IVF embryos exposed to exogenous vitamin C supplementation. Coinciding with rescued active DNA demethylation, we found that vitamin C significantly improved blastocyst formation and ICM specification, as well as implantation success and postimplantation survival of IVF embryos. A previous study showed that vitamin C supplementation in culture medium could reduce oxidative stress–induced embryo toxicity and improve the blastocyst development rate, thus the beneficial effect was thought to depend on its ROS-scavenging function as an antioxidant (Wang et al., 2002). In contrast, however, using chemical-induced inhibition of TET enzymatic activity, our results indicated that the effect of vitamin C on reversing IVF-induced impairment in DNA demethylation is largely mediated by TET enzymes. Of note, given previous studies have demonstrated TET1 and TET3 are main demethylase responsible for preimplantation active DNA demethylation (Ito et al., 2010; Gu et al., 2011; Kang et al., 2015) it is presumable that these two enzymes, rather than TET2, is primarily responsible for impaired active DNA demethylation in IVF embryos. In addition, the low-level vitamin C detected in oocytes and cumulus cells suggest that maternal deposit of vitamin C may also participate in active DNA demethylation during the period shortly after fertilization, perhaps mainly via the maternally deposited TET3.

Compared with notable efficacy of vitamin C, exogenous α-KG supplementation is less effective in enhancing TET enzymatic activity, or synergize with vitamin C, in IVF embryos, implying that endogenously synthesized α-KG may be sufficient for developmental requirement. Of note, our results were not completely in accordance to those reported by a recent study (Zhang et al., 2019). A possible explanation is variable TCA cycle metabolism among IVF embryos, since α-KG is an important intermediate metabolite.

Our results also highlight the important role of paracrine factors from maternal oviduct in fine-tuning epigenomic reprogramming during preimplantation development. Because vitamin C are enriched in both embryos and oviductal environment, it is presumable that TET enzymatic activity is well orchestrated via the synergic effects of embryonic autocrine and maternal paracrine. In addition, since the vitamin C levels are comparable between IVO and IVF embryos, the loss of oviductal vitamin C may be may be the main contributor to impaired TET enzymatic activity of IVF embryos. This concept, is in line with results reported by P Coy *et al*: DNA methylation and gene expression of IVF embryos can be partially corrected via supplementation of culture medium with oviductal fluid (Barrera et al., 2017; Canovas et al., 2017). Given safety concerns of transmission of diseases have not fully been addressed after addition of oviductal fluids, this strategy is only applicable to *in vitro* embryo production in domestic and laboratory animals. By contrast, the chemically defined culture medium that can specifically correct epigenetic errors in IVF embryos should be a more reasonable strategy, especially in the context of clinical use of human assisted reproductive technologies. Until now, however, only very limited growth factors or cytokines that present in oviductal fluid are proven to be used in commercially available culture media (Chronopoulou and Harper, 2015). Thus, identifying the developmental role of oviductal cytokines or growth factors in supporting early embryogenesis, and thus formulating the culture media, may be a promising strategy.

**FIGURE 7:**
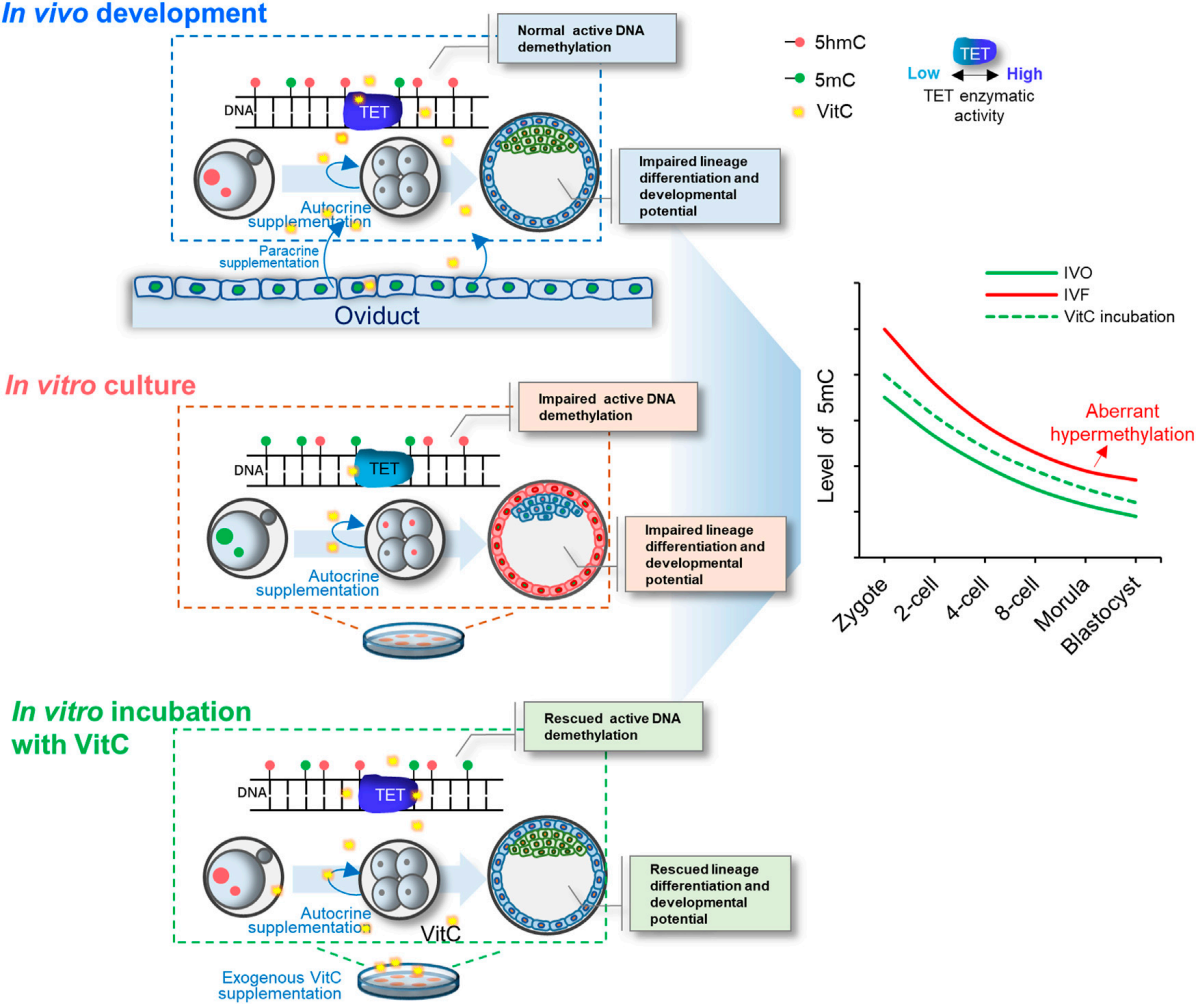
A model illustrating the important role of vitamin C in stimulating TET-mediated DNA demethylation in preimplantation embryos. Exogenous vitamin C supplementation into culture medium rescues impaired DNA demethylation in IVF embryos by enhancing TET enzymatic activity, and thus improving preimplantation lineage differentiation and promoting developmental potential of IVF blastocysts.

In summary, focusing on hypermethylated IVF blastocysts, our study identifies that TET-mediated DNA demethylation is impaired in IVF embryos throughout preimplantation development. Exogenous vitamin C supplementation into culture medium corrects DNA demethylation in IVF embryos by enhancing TET enzymatic activity, and thus improving preimplantation lineage differentiation and promoting developmental potential of IVF blastocysts (Figure 7). Our current findings have not only suggested a potential strategy for preventing or correcting IVF-associated epigenetic errors via the use of oviductal growth factors or cytokines, but also highlighted the important role of maternal oviduct in supporting embryonic epigenomic reprogramming.

## Data Availability

All relevant data is contained within the article. The original contributions presented in the study are included in the article/Supplementary Material, further inquiries can be directed to the corresponding author.
