# Supplementary material for: Vitamin C Rescues in vitro Embryonic Development by Correcting Impaired Active DNA Demethylation
Source: Front Cell Dev Biol. 2021 Nov 19;9:784244. doi: 10.3389/fcell.2021.784244 (PMC8640463; doi:10.3389/fcell.2021.784244)
Supplement: Supplementary file 3 [file DataSheet1.PDF]

Fig.S1

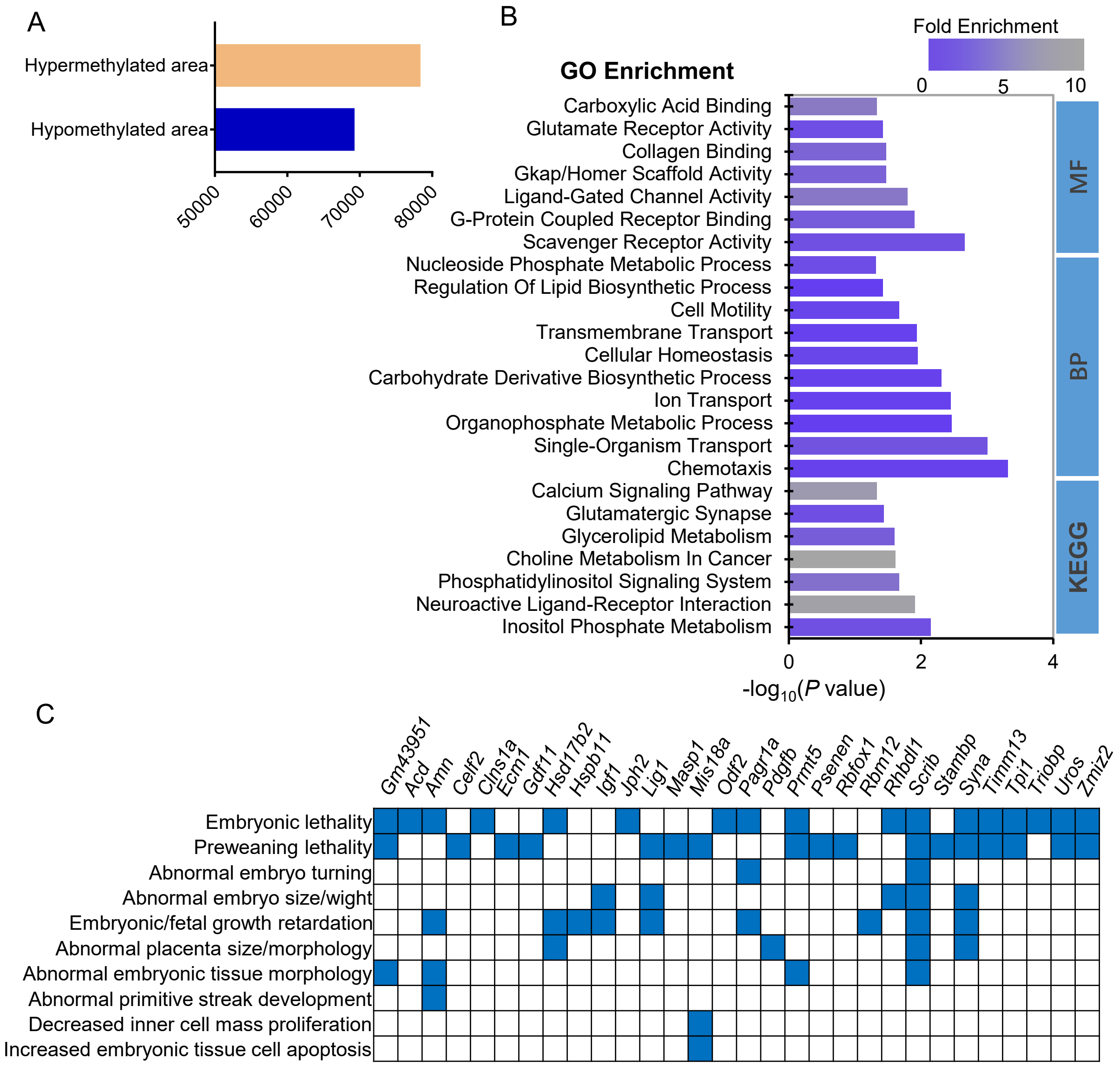
A
Hypermethylated area
Hypomethylated area
50000
60000
70000
80000
B
GO Enrichment
Fold Enrichment
0
5
10
Carboxylic Acid Binding
Glutamate Receptor Activity
Collagen Binding
Gkap/Homer Scaffold Activity
Ligand-Gated Channel Activity
G-Protein Coupled Receptor Binding
Scavenger Receptor Activity
Nucleoside Phosphate Metabolic Process
Regulation Of Lipid Biosynthetic Process
Cell Motility
Transmembrane Transport
Cellular Homeostasis
Carbohydrate Derivative Biosynthetic Process
Ion Transport
Organophosphate Metabolic Process
Single-Organism Transport
Chemotaxis
Calcium Signaling Pathway
Glutamatergic Synapse
Glycerolipid Metabolism
Choline Metabolism In Cancer
Phosphatidylinositol Signaling System
Neuroactive Ligand-Receptor Interaction
Inositol Phosphate Metabolism
MF
BP
KEGG
0
2
4
-log10(P value)
C
Gm43951
Acd
Amn
Celf2
Clns1a
Ecm1
Gdf11
Hsd17b2
Hspb11
Igf1
Jph2
Lig1
Masp1
Mis18a
Odf2
Pagr1a
Pdgfb
Prmt5
Psenen
Rbfox1
Rbm12
Rhbdl1
Scrib
Stambp
Syna
Timm13
Tpi1
Triobp
Uros
Zmiz2
Embryonic lethality
Preweaning lethality
Abnormal embryo turning
Abnormal embryo size/wight
Embryonic/fetal growth retardation
Abnormal placenta size/morphology
Abnormal embryonic tissue morphology
Abnormal primitive streak development
Decreased inner cell mass proliferation
Increased embryonic tissue cell apoptosis

Fig.s2

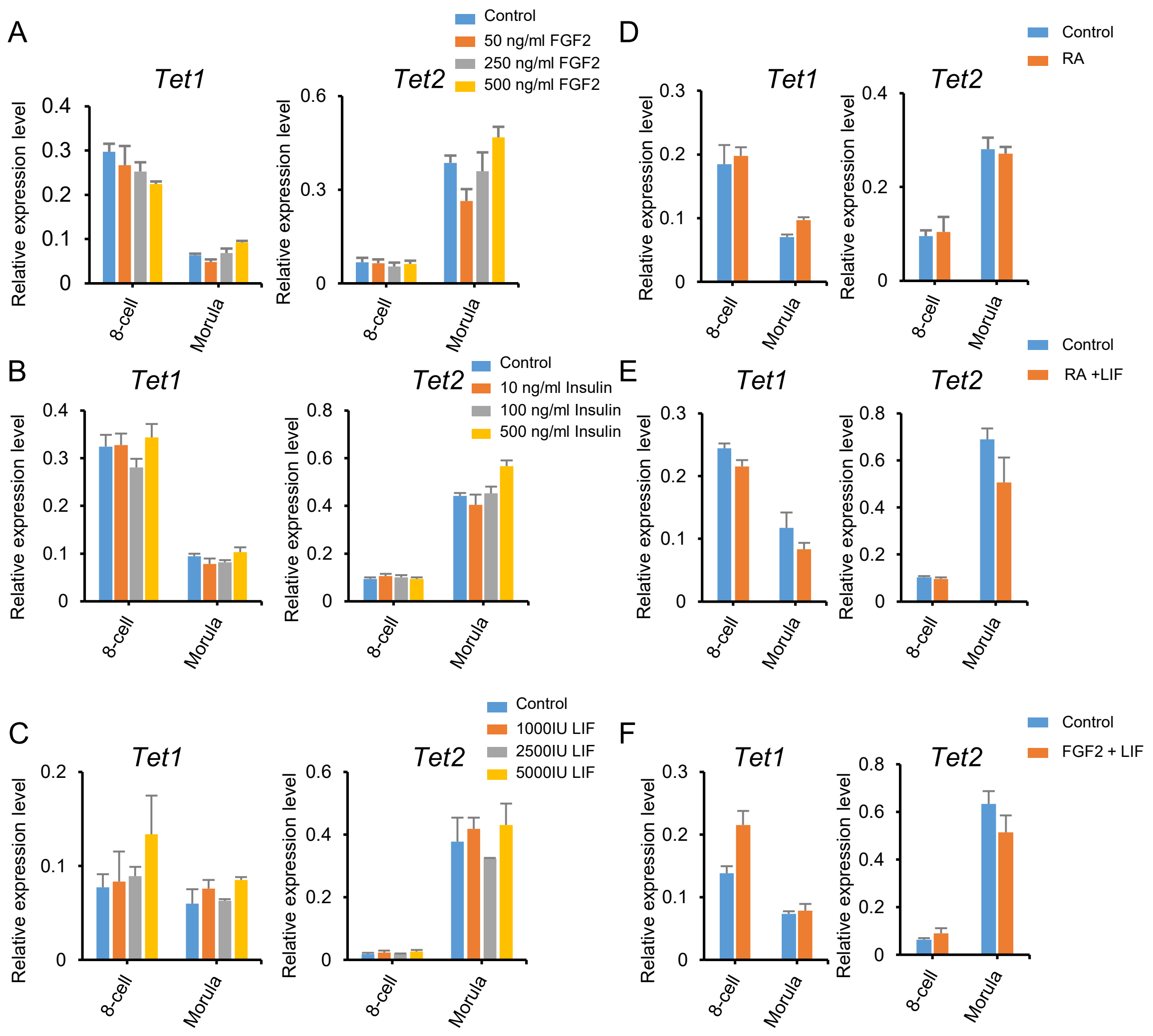
A
Tet1
Tet2
Control
50 ng/ml FGF2
250 ng/ml FGF2
500 ng/ml FGF2
B
Tet1
Tet2
Control
10 ng/ml Insulin
100 ng/ml Insulin
500 ng/ml Insulin
C
Tet1
Tet2
Control
1000IU LIF
2500IU LIF
5000IU LIF
D
Tet1
Tet2
Control
RA
E
Tet1
Tet2
Control
RA +LIF
F
Tet1
Tet2
Control
FGF2 + LIF
Relative expression level
8-cell
Morula

Fig.s3

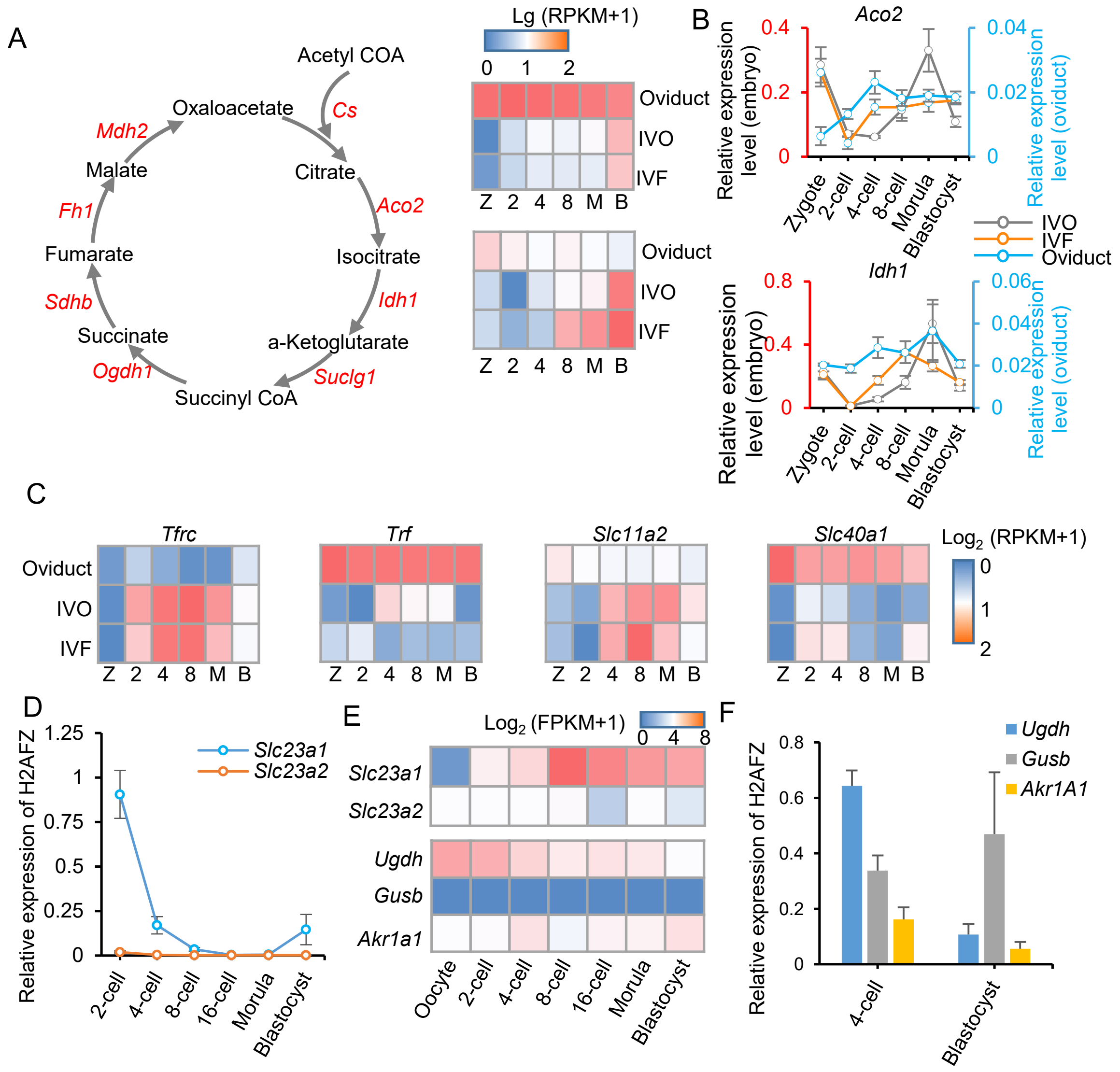

A
Acetyl COA
Cs
Oxaloacetate
Citrate
Aco2
Isocitrate
Idh1
a-Ketoglutarate
Suclg1
Succinyl CoA
Ogdh1
Succinate
Sdhb
Fumarate
Fh1
Malate
Mdh2
Lg (RPKM+1)
0 1 2
Oviduct
IVO
IVF
Z 2 4 8 M B
B
Aco2
Relative expression level (embryo)
Relative expression level (oviduct)
0.4 0.2 0
0.04 0.02 0
Zygote 2-cell 4-cell 8-cell Morula Blastocyst
IVO
IVF
Oviduct
Idh1
0.8 0.4 0
0.06 0.04 0.02 0
C
Tfrc
Trf
Slc11a2
Slc40a1
Log2 (RPKM+1)
0 1 2
D
Relative expression of H2AFZ
1.25 1 0.75 0.5 0.25 0
Slc23a1
Slc23a2
2-cell 4-cell 8-cell 16-cell Morula Blastocyst
E
Log2 (FPKM+1)
0 4 8
Slc23a1
Slc23a2
Ugdh
Gusb
Akr1a1
Oocyte 2-cell 4-cell 8-cell 16-cell Morula Blastocyst
F
Relative expression of H2AFZ
0.8 0.6 0.4 0.2 0
Ugdh
Gusb
Akr1A1
4-cell
Blastocyst

Fig. s4

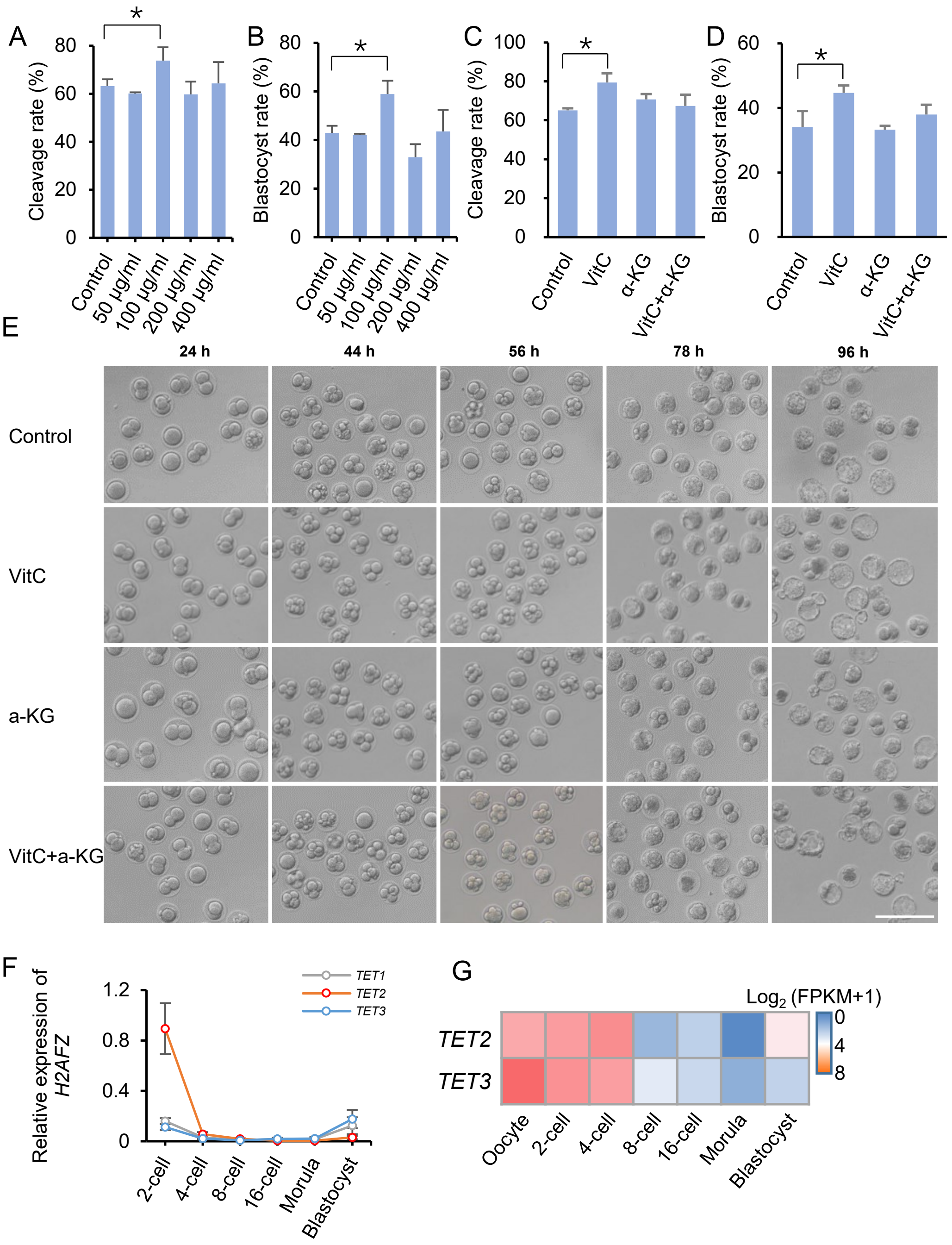

A
Cleavage rate (%)
0
20
40
60
80
*
Control
50 μg/ml
100 μg/ml
200 μg/ml
400 μg/ml
B
Blastocyst rate (%)
0
20
40
60
80
*
Control
50 μg/ml
100 μg/ml
200 μg/ml
400 μg/ml
C
Cleavage rate (%)
0
20
40
60
80
100
*
Control
VitC
α-KG
VitC+α-KG
D
Blastocyst rate (%)
0
20
40
60
*
Control
VitC
α-KG
VitC+α-KG
E
24 h
44 h
56 h
78 h
96 h
Control
VitC
a-KG
VitC+a-KG
F
Relative expression of H2AFZ
0
0.4
0.8
1.2
TET1
TET2
TET3
2-cell
4-cell
8-cell
16-cell
Morula
Blastocyst
G
Log2 (FPKM+1)
0
4
8
TET2
TET3
Oocyte
2-cell
4-cell
8-cell
16-cell
Morula
Blastocyst

Table s1. Sequences of quantitative PCR primers

	Gene Name	Sense (5′ to 3′)	Antisense (5′ to 3′)
**Mouse**	*Gapdh*	TGCCCCCATGTTTGTGATG	TGTGGTCATGAGCCCTTCC
	Tet1	ATTTCCGCATCTGGGAACCTG	GGAAGTTGATCTTTGGGGCAAT
	Tet2	AGAGAAGACAATCGAGAAGTCGG	CCTTCCGTACTCCCAAACTCAT
	Tet3	TGCGATTGTGTCGAACAAATAGT	TCCATACCGATCCTCCATGAG
	Slc2a1	CCTCGTGCTCTTCTTCATCTT	CTCGGGTGTCTTGTCACTTT
	Slc23a1	TGGAGATCTCAGCTCTACCTATT	GAATGCAGTCCAGGTCTCTTAG
	Slc23a2	GTGTCTCGCTTGTGTCCTTAT	ATGGCAGGGCTGTGATTT
	Ugp2	GACTGGTGGAAATCGCTCAA	CTGCTCCAAGAGAAATCCATAGG
	Ugdh	CATCGGGATGGACCAAAGAA	CAGAGCCTCACAGAGATAAACC
	Gusb	TCGATCTGTGGTCCTCTATACC	CCCTGCACAGAAATCCAGTAG
	Akr1A1	GCTTGGAGGTGACTGCTTAT	CTAGTGCCAAGACTACTGGTTC
	Gulo	CACCTTCTGGAGACATCCTTTC	GGTAGATGATGCTGACGTTCTC
	Rgn	AGCCATGGTGGATGAAGATAAG	GCCATGGTACCAGCAAAGTA
	Aco2	CAAGTATGGTGTGGGCTTCT	GATCAGAAGAACTCCAGGGTATG
	Idh1	GGGCATGTACAACCAGGATAA	AGTGTTCTTGGTGCTGAGATAC
**Bovine**	*Slc23A1*	CAGGAAGCCCAGAAGAACGA	TGAAGACCGGGCAGATAGGA
	Slc23A2	GCCATGTGTGTGGGGTATGA	GGGCCAGAAATGCAAAAGCA
	Ugdh	TGCCTGGAAGTGGTACAGTC	GAGTTCCATGCATTGATTCTTGA
	Gusb	CAGCAACGTGTCTCTGCAAC	TTAGTGTGGGCAATCAGCGT
	Akr1A1	ACCCTGTTAACGCTCTGTACC	GCCTACACTCAGGGCATACT
	Tet1	CGACCAAAACCTCGTGCAAC	CGTGGCGAGATCGAGACATA
	Tet2	TTCCGAACCCACTTACCTGC	TAACCAACTCAAGGGGCGAC
	Tet3	GTGACCCTGCAGCAGAGATT	TTGAGGGCTGCTCTTTGGAG
	H2afz	CTCACCGCAGAGGTACTTGAATT	AGTCCAATTCTTCATCTCCACGA
